# TG/HDL-C ratio predicts in-hospital mortality in patients with acute type A aortic dissection

**DOI:** 10.1186/s12872-022-02793-5

**Published:** 2022-08-01

**Authors:** Yan-Juan Lin, Jian-Long Lin, Yan-Chun Peng, Sai-Lan Li, Liang-Wan Chen

**Affiliations:** 1 Department of Nursing, Fujian Medical University Union Hospital, No. 29 Xinquan Road, Fuzhou, 350001 Fujian China; 2 Department of Cardiac Surgery, Fujian Medical University Union Hospital, No. 29 XinquanRoad, Fuzhou, 350001 Fujian China

**Keywords:** Type A acute aortic dissection, In-hospital mortality, TG/HDL-C ratio, Outcome

## Abstract

**Background:**

In recent years, abnormalities in serum lipids and lipoproteins have been shown to be associated with cardiovascular disease risk. However, their prognostic value for acute type A aortic dissection is unclear. This study analyzed the correlation between triglyceride/high-density lipoprotein cholesterol (TG/HDL-C) ratio and in-hospital mortality in patients with AAAD, and aimed to investigate the clinical significance of preoperative blood lipids and lipoproteins on the prognosis of acute type A aortic dissection.

**Methods:**

A total of 361 patients who underwent type A aortic dissection surgery in Fujian Cardiac Medical Center from June 2018 to March 2020 were retrospectively collected. According to the baseline TG/HDL-C ratio, the patients were divided into 3 groups according to the tertile method, the low TG/HDL-C ratio T1 group (< 1.18) and the middle TG/HDL-C ratio T2 group (1.18–1.70). T3 group with high TG/HDL-C ratio (> 1.70). Kaplan–Meier was used for survival analysis, and Cox proportional hazards regression model was used to analyze the factors affecting the prognosis of patients. The receiver operating characteristic (ROC) curve was used for the diagnostic efficacy.

**Results:**

Among the 361 patients in this study, the mean age was 52.4 ± 11.3 years, 73 (20.2%) were female, and 82 (22.7%) died in hospital. Kaplan–Meier curve showed that with the increase of TG/HDL-C ratio, the risk of in-hospital death gradually increased (*P* < 0.001). Multivariate Cox regression analysis showed that age (HR = 1.031), body mass index (HR = 1.052), hypertension (HR = 3.491), white blood cells (HR = 1.073), TG/HDL-C ratio (HR = 1.604), MODS (HR = 1.652) was positively correlated with in-hospital mortality (*P* < 0.05). After adjusting for age, sex, and other risk factors, a significant association was found between the TG/HDL-C ratio and in-hospital mortality for acute type A aortic dissection (HR = 1.472, 95% CI, 1.354–3.451, *P* = 0.019).

**Conclusion:**

Patients with type A aortic dissection have obvious abnormal blood lipid metabolism, and serum TG/HDL-C levels are positively correlated with in-hospital mortality in patients with AAAD.

## Key messages

1. With the increase of TG/HDL-C ratio, the risk of in-hospital death gradually increased
2. This study indicated 6 risk factors were positively correlated with in-hospital mortality.
3. The serum TG/HDL-c ratio may help guide the treatment and prevention of acute type A aortic dissection.

## Background

Acute aortic dissection (AAD) is a potentially fatal emergency macrovascular disease. Clinically, it is classified into Stanford type A and B according to whether the dissection involves the root of the artery, the ascending aorta, or the aortic arch. According to statistics, the incidence of AAD in China is 2.78/100,000 [1]. Among them, type A aortic dissection (AAAD) is the main AAD, and its incidence accounts for about 2/3 of the total AAD [2]. In recent years, the incidence and mortality of AAAD have remained high. With the increase in the proportion of hypertension, the incidence of AAAD has been increasing year by year. At present, timely surgical treatment is the main effective method for AAAD, but the early mortality after AAAD is still as high as 8–25% [3, 4]. Therefore, early identification of risk factors for death on admission in AAD patients is of great significance to reduce mortality and improve prognosis.

Previous studies have reported that there is a certain correlation between laboratory test indicators such as uric acid, creatinine, C-reactive protein, D-dimer, and fibrinogen and in-hospital mortality in AAD patients [5]. These biomarkers can predict early postoperative mortality in AAAD to some extent. However, there are few reports on the relationship between blood lipid levels and AAAD at admission.

Abnormal blood lipids and lipoproteins are one of the important markers of cardiovascular disease. Triglyceride and high-density lipoprotein, as important blood lipid and lipoprotein components in the human body, play an important role in the pathogenesis and prognosis of cardiovascular diseases such as hypertension, coronary artery disease, heart failure and atrial fibrillation [6]. The ratio of triglyceride to high-density lipoprotein cholesterol (TG/HDL-C) can reflect the comprehensive level of lipid metabolism in the body, and it has been confirmed to be a risk factor for various cardiovascular diseases [7]. However, the application of lipid metabolism in AAAD and its effect on prognosis are less.

AAD is often accompanied by dyslipidemia and lipoprotein abnormalities. In different types of AAD, the level of triglyceride increased to different degrees, and the level of high-density lipoprotein cholesterol decreased to different degrees [8]. This suggests that triglyceride and high-density lipoprotein cholesterol levels have a certain relationship with the incidence and prognosis of AAD. It has been reported that in type B AAD, TG/HDL-C is inversely correlated with patient mortality, and its ratio can be used to predict the postoperative risk of type B AAD [9]. However, there is no clinical correlation study between TG/HDL-C and early postoperative mortality after AAAD.

Therefore, this study conducted a retrospective study of 361 patients who underwent surgery for type A aortic dissection. The association of triglyceride/high-density lipoprotein cholesterol (TG/HDL-C) ratio at admission with in-hospital mortality in AAAD patients was investigated by survival analysis and Cox proportional hazards regression model. To provide a reference for finding clinical indicators that are highly correlated with the early mortality of AAAD, real and easy to detect, and has important clinical significance for reducing the risk of surgery and reducing the early mortality after AAAD.

## Materials and methods

### Patient selection

**Fig. 1:**
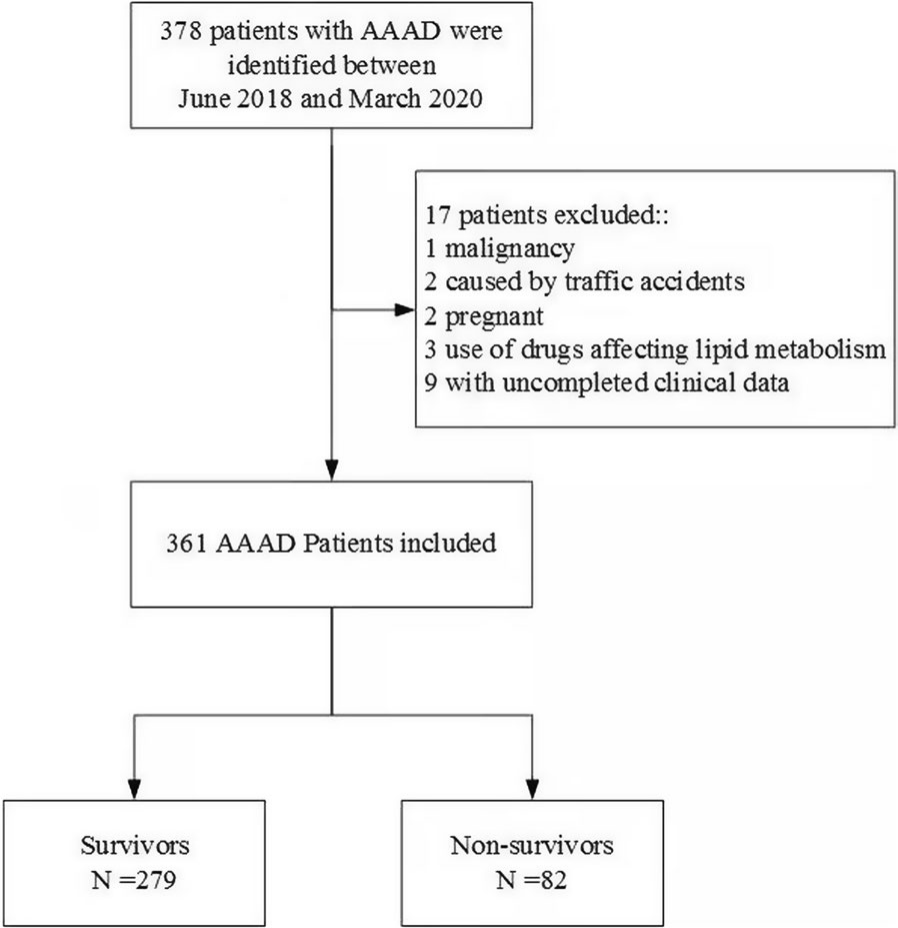
The subjects included in this study

This study was a retrospective observational study. A total of 378 patients with type A aortic dissection who were diagnosed by computed tomography angiography (CTA) and magnetic resonance imaging (MRI) and underwent surgery for type A aortic dissection from June 2018 to March 2020 in Fujian Cardiac Medical Center were collected. Inclusion criteria: AAAD surgery patients aged ≥ 18 years, the diagnostic criteria refer to the 2014 ESC Diagnosis and Treatment of Aortic Diseases [10]. Exclusion criteria: (1) patients with trauma-induced AAAD and pregnant women; (2) complicated with heart failure, liver insufficiency and other important organ damage diseases; (3) long-term use of drugs that may affect blood results; (4) Patients without full medical records. Finally, 361 AAAD patients were included in the study (Fig. 1). This study was approved by the hospital ethics committee, ethics committee approval number: 2020KY082. This study was a retrospective observational study without informed consent. Consistent with the Helsinki Declaration.

All the patients were admitted to the intensive care unit and were given sedation, analgesia, oxygen inhalation, and keep a normal bowel movement. Urapidil and sodium nitroprusside were used to control the systolic blood pressure within 100–120 mmHg (1 mmHg = 0.133 kPa), and β-receptor blockers were used to control the heart rate within 60–80/min. We researched AAAD patients with cear diagnosis by CTA and almost all of them have undergone implantation of modified triple-branched stent graft for descending aorta replacement in addition to aortic root reconstruction and ascending aorta or hemiarch replacement. The rest in Stanford type B were treated with thoracic endovascular aortic repair (TEVAR).

### Clinical assessment

Patient data were collected through an electronic medical record system, demographic characteristics including age, sex, body mass index (BMI); past history, including hypertension, diabetes, coronary heart disease, smoking history, drinking history; Vital signs on admission, including systolic blood pressure, diastolic blood pressure, and heart rate; Laboratory tests, including white blood cells, platelets, triglycerides, high-density lipoprotein cholesterol, fasting blood glucose, C-reactive protein, serum creatinine, blood urea nitrogen; The patient's treatment regimen and prognosis during hospitalization were recorded. The TG/HDL-C ratio is calculated by dividing the TG and HDL-C values. The primary outcome measure was in-hospital mortality, and the secondary outcome measure was postoperative complications, including pulmonary infection, acute renal failure, arrhythmias, and multiple organ dysfunction (MODS). The number of hospital days in the intensive care unit (ICU) was also recorded. Acute renal failure was defined as a creatinine level 2 times the preoperative creatinine or requiring renal replacement therapy.

### Statistical analysis

Measurement data were described as "mean ± standard deviation" (normal distribution), median and interquartile range (IQR) (skewed distribution), and categorical variables were expressed as frequencies or percentages. According to the TG/HDL-C variable, the cut points were divided into three equal parts (T1, T2, T3), and the Kruskal–Wallis H (skewed distribution) and One-Way ANOVA test (normal distribution) were used for comparison between groups; count data were compared using χ^2^ test. Cox proportional hazards regression model was used to analyze the factors affecting the prognosis of patients, and Kaplan–Meier curves were used to compare the relationship between TG/HDL-C ratio and in-hospital mortality. After adjusting for other potential confounding factors, different models were constructed to examine the independent effect of TG/HDL-C ratio on in-hospital mortality. Finally, subgroup analysis was performed on age, BMI, hypertension classification, MODS, etc. The receiver operating characteristic (ROC) curve was used for the diagnostic efficacy. The area under curve (AUC), sensitivity, and specificity were calculated respectively to determine the value of TG, HDL-C and TG/HDL-C in the prediction of in-hospital mortality of AAAD patients. Data processing was performed using R 4.1.1 software. Differences were considered statistically significant at *P* < 0.05.

## Result

### Clinical features

A total of 361 AAAD patients were included in this study, including 288 males and 73 females, with an average age of 50.2 ± 7.1 years, ranging in age from 36 to 69 years. All patients underwent AAAD surgery. Dissection involved the ascending aorta in 103 cases (28.5%), combined the aortic arch in 34 cases (9.4%), combined the thoracic aorta in 96 cases (26.6%), and combined the abdominal aorta in 128 cases (35.5%). Comparison of baseline characteristics of subjects with different TG/HDL-C levels in three groups (Table 1): Compared with (T1 and T2), patients in high TG/HDL (T3) group had significantly higher white blood cell counts (*P* < 0.001), higher body mass index (*P* = 0.019), increased age (*P* = 0.020), prolonged operation time (*P* = 0.008), hepatic insufficiency (*P* = 0.047) and prolonged ICU stay (*P* = 0.003). The remaining variables were not statistically different among the groups (*P* > 0.05). Compared with low TG/HDL (T1), AAAD patients in the high TG/HDL (T3) group had the highest in-hospital mortality (40%) (*P* < 0.01).

**Table 1:** Comparison of baseline data in different TG/HDL-C ratio groups

Variable	TG/HDL-C ratio (mmol/L)(tertile)			*P* value
	T1 (< 1.18) N = 121	T2 (1.18–1.70) N = 120	T3 (> 1.70) N = 120	
Age (years)	49.5 ± 6.2	49.4 ± 6.3	51.7 ± 8.4	0.020
Female n, (%)	25 (20.7)	22 (18.3)	26 (21.7)	0.805
BMI (kg/m^2^)	24.0 ± 3.2	24.8 ± 3.4	25.3 ± 4.3	0.019
**Physical examination**				
SBP	150.4 ± 27.3	146.9 ± 28.5	149.2 ± 24.9	0.596
DBP	77.7 ± 15.6	77.8 ± 16.8	74.5 ± 18.5	0.240
Heart rate	85.1 ± 15.3	80.6 ± 15.3	83.0 ± 16.4	0.083
Smoker n, (%)	63 (52.1)	75 (62.5)	67 (55.8)	0.254
Drinker n, (%)	57 (47.1)	66 (55.0)	64 (53.3)	0.433
**Medical history**				
Diabetes mellitusn, (%)	16 (13.2)	16 (13.3)	17(14.2)	0.973
Hypertensionn, (%)	111 (91.7)	112 (93.3)	104 (86.7)	0.182
Atherosclerosisn, (%)	2 (1.7)	2 (1.7)	4 (3.3)	0.596
Marfan's syndrome n, (%)	3(2.5)	3 (2.5)	1 (0.8)	0.556
**Imaging examination**				
Aortic insufficiency n, (%)	15 (12.4)	23 (19.2)	25 (20.8)	0.188
LVEF (%)	65 (58,70)	64 (56,68)	64 (58,69)	0.407*
**Laboratory results**				
WBC (× 10^9^/L)	9.09 ± 3.07	9.34 ± 3.02	11.05 ± 4.04	< 0.001
PLT (× 10^9^ /L)	152 (125, 196)	154 (124, 199)	162 (128, 191)	0.945*
Hb (g/L)	125.17 ± 16.18	125.47 ± 21.11	126.40 ± 20.25	0.875
Blood glucose (mmol/L)	6.0 (5.2, 7.7)	6.5 (5.4, 7.7)	6.1 (5.4, 7.9)	0.623*
Cr (mmol/L)	91 (69, 133)	86 (74, 127)	96 (71, 130)	0.805*
BUN (mmol/L)	6.5 (4.8, 8.7)	6.2 (4.8, 8.7)	6.4 (4.8, 8.9)	0.948*
TG (mmol/L)	1.36 ± 0.57	1.63 ± 0.54	2.17 ± 0.92	< 0.001
HDL-c (mmol/L)	1.70 ± 0.92	1.08 ± 0.37	0.89 ± 0.29	< 0.001
Operation time (min)	308 (274, 340)	295 (250, 350)	310 (270, 385)	0.028*
CPB time (min)	160 (135, 189)	140 (129, 183)	154 (133, 224)	0.056*
Aortic clamping time (min)	72 (54, 105)	65 (49, 91)	78 (62, 98)	0.051*
ICU stay (day)	7 (4, 10)	8 (5, 14)	8 (5, 13)	0.022*
Hospital stay (day)	23 (17, 34)	21 (16, 30)	22 (17, 32)	0.521*
**Complications**				
Liver insufficiency	23 (19.0)	13 (10.8)	33 (27.5)	0.005
Cardiac tamponed	1 ( 0.8)	7 ( 5.9)	6 ( 5.0)	0.098
Acute kidney injury	28 (23.1)	29 (24.2)	32 (26.7)	0.808
Death in hospital	6 (5.0)	28 (23.3)	48 (40.0)	< 0.001

Data are median (quartile25%-quartile75%) or N (%)

*BMI* body mass index, *SBP* systolic blood pressure, *DBP* diastolic blood pressure, *LVEF* left ventricular ejection fraction, *WBC* white blood cell count, *PLT* platelet count, *Hb* hemoglobin, *Cr* creatinine, *BUN* blood urea nitrogen, *TG* triglyceride, *HDL-c* high-density lipoprotein, *CPB* cardiopulmonary bypass time, *ICU* intensive care unit

^*^Kruskal–Wallis H

### Cox regression analysis of factors affecting the prognosis of patients after AAAD

Univariate Cox regression analysis of in-hospital deaths in AAAD patients showed that age, body mass index, hypertension, diabetes, systolic blood pressure on admission, white blood cells, TG/HDL-C ratio, cardiopulmonary bypass time, MODS, postoperative renal damage, Postoperative pneumonia is a risk factor for in-hospital mortality. Multivariate regression analysis showed that age, body mass index, the presence or absence of hypertension, white blood cells, TG/HDL-C ratio, MODS were significantly associated with in-hospital mortality (*P* < 0.05) (Table 2).

**Table 2:** Cox proportional hazards regression analysis for type A aortic dissection

Variable	Univariate model		Multivariate model	
	*OR* (95%CI)	*P* value	*OR* (95%CI)	*P* value
Age(years)	1.005 (1.029–1.082)	< 0.001	1.031 (1.003–1.060)	0.029
Gender	1.053 (0.601–1.846)	0.856	–	–
BMI(kg/m^2^)	1.094 (1.039–1.152)	0.001	1.052 (0.881–0.974)	0.045
**Physical examination**				
SBP	1.012 (1.003–1.000)	0.008	1.000 (0.988–1.011)	0.961
DBP	1.000 (0.988–1.013)	0.942	–	–
Heart rate	0.994 (0.980–1.007)	0.358	–	–
Smoker	1.002 (0.644–1.558)	0.993	–	–
Drinker	1.007 (0.652–1.555)	0.975	–	–
**Medical history**				
Diabetes mellitus	1.677 (1.020–2.756)	0.041	1.608 (0.919–2.813)	0.096
Hypertension	1.756 (1.031–2.992)	0.038	3.491 (1.573–7.749)	0.002
Cerebral infarction	1.266 (0.463–3.465)	0.646	–	–
Atherosclerosis	1.168 (0.367–3.713)	0.792	–	–
Marfan's syndrome	1.094 (0.151–7.933)	0.929	–	–
**Imaging examination**				
Aortic insufficiency	1.070 (0.627–1.828)	0.804	–	–
LVEF (mm)	0.998 (0.976–1.021)	0.877	–	–
**Laboratory results**				
WBC (× 10^9^/L)	1.132 (1.077–1.191)	< 0.001	1.073 (1.015–1.135)	0.013
PLT (× 10^9^ /L)	0.998 (0.994–1.001)	0.197	–	–
Hb (g/L)	0.993 (0.982–1.003)	0.176	–	–
Blood glucose (mmol/L)	0.970 (0.905–1.041)	0.403	–	–
Cr (mmol/L)	1.000 (0.998–1.002)	0.749	–	–
BUN (mmol/L)	1.031 (0.986–1.077)	0.176	–	–
TG (mmol/L)	1.417 (1.133–1.772)	0.002	–	–
HDL-c (mmol/L)	0.594 (0.371–0.951)	0.030	–	–
TG/HDL-C ratio (mmol/L)	1.907 (1.517–2.398)	0.001	1.604 (1.223–2.104)	0.001
Operation time (min)	1.002 (0.999–1.004)	0.133	–	–
CPB time (min)	1.004 (1.000–1.007)	0.025	1.001 (0.997–1.005)	0.538
Aortic clamping time (min)	1.004 (0.999–1.008)	0.118	–	–
**In-hospital complications**				
MODS	1.745 (1.115–2.731)	0.015	1.652(1.034–2.640)	0.036
Liver insufficiency	1.225 (0.733–2.047)	0.439	–	–
Acute kidney injury	1.916 (1.207–3.041)	0.006	1.629(0.977–2.718)	0.061
Gastrointestinal bleeding	1.621 (0.504–5.217)	0.418	–	–
Pneumonia	1.894 (1.002–3.581)	0.049	1.020(0.478–2.173)	0.960
Intubation	1.012 (0.464–2.205)	0.977	–	–

*BMI* body mass index, *SBP* systolic blood pressure, *DBP* diastolic blood pressure, *LVEF* left ventricular ejection fraction, *WBC* white blood cell count, *PLT* platelet count, *Hb* hemoglobin, *Cr* creatinine, *BUN* blood urea nitrogen, *TG* triglyceride, *HDL-c* high-density lipoprotein, *CPB* cardiopulmonary bypass time, *MODS* multiple organ failure, *HR* hazard ratio, *CI* confidence interval

### Survival curve of patients after AAAD with different TG/HDL-C levels

**Fig. 2:**
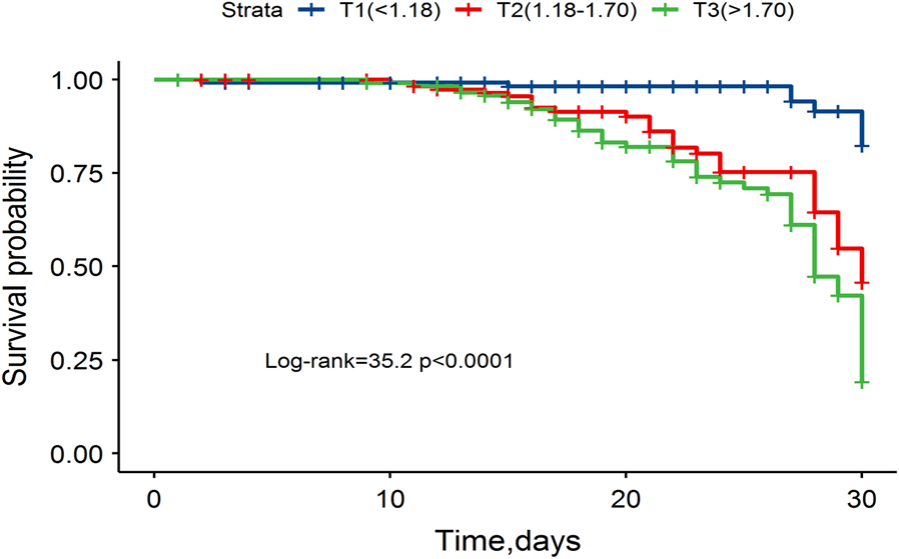
Kaplan–Meier in-hospital mortality analysis

Kaplan–Meier analysis showed that the cumulative survival rate of different TG/HDL-C levels was also significantly different (log-rank χ^2^ = 35.2, *P* < 0.001). The cumulative in-hospital survival rate of the T1 group was significantly higher than that of the other groups (Fig. 2).

### Relationship between TG/HDL-C ratio and in-hospital mortality

Different models were constructed to analyze the independent effect of TG/HDL-C ratio on in-hospital mortality using Cox proportional hazards models after adjusting for confounding variables (Table 3). The results showed that the TG/HDL-C ratio (HR = 1.907, 95% CI, 1.517–2.398, *P* = 0.001) remained an important predictor of in-hospital mortality. In an unadjusted model (rough model), TG/HDL-C was positively associated with in-hospital mortality (HR = 1.907, 95% CI = 1.517–2.398, *P* = 0.001). In addition, in-hospital mortality in the T3 group was 8.675 times higher than that in the T1 group (HR = 8.675, 95% CI, 3.705–20.312; *P* = 0.001). In Model I (adjusted for age, sex, and body mass index), in-hospital mortality was 7.077 times higher in the T3 group than in the T1 group (HR = 7.077, 95% CI, 2.991–16.745; *P* = 0.001). In Model II (adjusted for covariates such as age, sex, body mass index, hypertension, diabetes, cerebral infarction, smoking, alcohol consumption, atherosclerosis, white blood cell count, hemoglobin, platelet count, blood urea nitrogen, creatinine, etc.), group T3 The in-hospital mortality rate was 2.105 times higher than that in the T1 group (HR = 2.105, 95% CI, 1.709–14.273; *P* = 0.004). For the purpose of sensitivity analysis, we treated the continuous variable (TG/HDL-C) as three equal categorical variables. The P values for the TG/HDL-C trend were consistent with the results when TG/HDL-C was treated as a continuous variable.

**Table 3:** In-hospital mortality risk association in different TG/HDL-C ratio groups

	HR (95% CI), *P* value		
	Crude model	Model I	Model II
TG/HDL-C ratio	1.907 (1.517–2.398), 0.001	1.741(1.366–2.219), 0.001	1.472(1.354–3.451), 0.019
**TG/HDL-C ratio**			
T1 (< 1.18)	Ref	Ref	Ref
T2 (1.18–1.70)	5.306(2.191–12.848), 0.001	5.109(2.107–12.389), 0.001	3.557(1.196–10.579), 0.023
T3 (> 1.70)	8.675(3.705–20.312), 0.001	7.077(2.991–16.745), 0.001	2.105(1.709–14.273), 0.004
*P* for trend	< 0.001	< 0.001	0.002

### Subgroup analysis

**Fig. 3:**
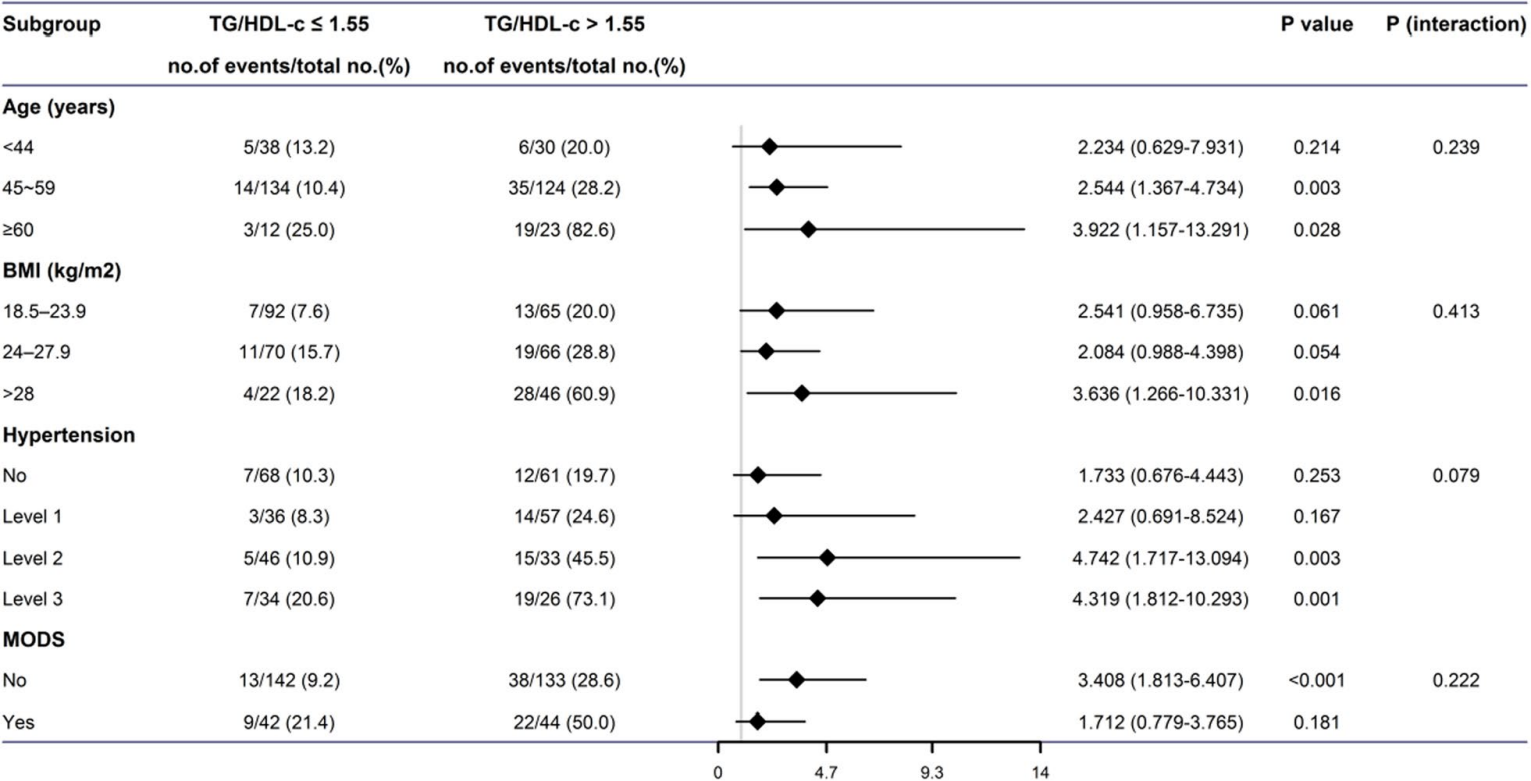
Subgroup analysis of in-hospital mortality

Subgroup analysis of in-hospital mortality was performed by age, body mass index, hypertension classification, and MODS. Compared with the low TG/HDL-C ratio group, in-hospital death was significantly higher in the group with high TG/HDL-C ratio than in the group with high TG/HDL-C ratio (*P* < 0.05). (Fig. 3).

### Sensitivity and specificity of TG, HDL-C and TG/HDL-C ratio in predicting in-hospital mortality

**Fig. 4:**
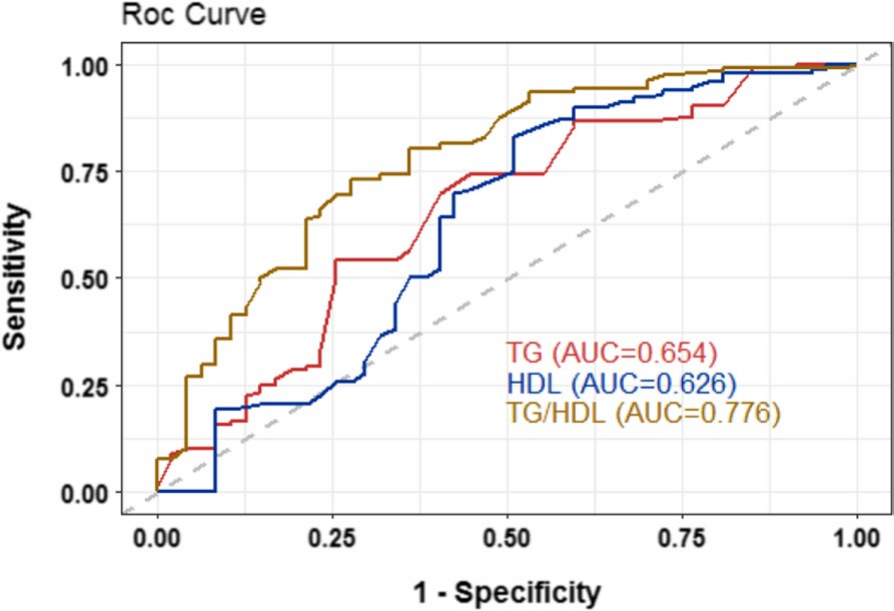
The AUC value of TG, HDL-C and TG/HDL-C for predicting in-hospital mortality

ROC analysis was used to determine possible cutoff values for TG, HDL-C, and TG/HDL-C ratios in predicting in-hospital mortality. It has been observed that TG/HDL-C can be used as an important indicator to predict in-hospital mortality in patients with AAAD. The AUC was 0.776 (95% CI: 0.627–0.864), the optimal cutoff value was1.404, the sensitivity was 72.9%, and the specificity was 72.3% (Fig. 4, Table 4).

**Table 4:** Diagnostic value of TG、HDL-C and TG/HDL-C for in-hospital mortality

	AUC	Cut-off value	95% CI	Sensitivity (%)	Specificity (%)
TG (mmol/L)	0.654	1.530	0.542–0.766	74.2	55.3
HDL-c (mmol/L)	0.626	0.890	0.447–0.805	83.1	48.9
TG/HDL-C	0.776	1.404	0.627–0.864	72.9	72.3

*AUC* area under the curve, *CI* confidence interval

## Discussions

Acute aortic dissection (AAD) is a fatal cardiovascular disease and urgent surgical treatment is the main strategy for AAAD [11]. Although its clinical prognosis has been greatly improved due to early diagnosis and prompt surgical management. However, its early mortality and long-term reoperation rate are still high, mainly due to the critical condition and complicated operation. If AAAD can be effectively assessed and predicted early, and adequate preparations are made, its mortality can be reduced [12]. The in-hospital mortality rate of patients in this study was 22.7%, which was lower than the 27.4% reported in Western countries [13]. The possible reason is that the age of the patients in this study (52.4 ± 11.3) was lower than that of the patients in the western study (61.5 ± 14.6), and the younger patients had better physical fitness and health prognosis. In addition, this study is a single-center study with a small sample size, and a multi-center study can be conducted in the future.

The results of multivariate regression analysis in this study showed that among patients undergoing AAAD surgery, age, body mass index (BMI), presence or absence of hypertension, white blood cells, TG/HDL-C ratio, and MODS were independent risk factors for in-hospital death in patients undergoing AAAD surgery. In addition, this study evaluated the relationship between the TG/HDL-C ratio at admission and postoperative in-hospital mortality in 361 AAD patients. The results showed that when AAD patients had a higher TG/HDL-C ratio on admission, their risk of postoperative in-hospital mortality was significantly increased. The TG/HDL-C ratio was significantly associated with in-hospital mortality for acute type A aortic dissection even after adjusting for age, sex, and other confounding factors.

The results of this study showed that age was an independent risk factor for postoperative in-hospital mortality in patients with AAAD. Consistent with previous findings, advanced age is an important risk factor for aortic dissection. Patients with aortic dissection over the age of 70 have a significantly higher probability of death in the acute phase than patients with aortic dissection at a younger age [14]. Elderly patients have low organ physiological reserve and poor tolerance to surgical shock and circulatory arrest. In addition, the elderly often have underlying diseases such as hypertension, diabetes, cerebrovascular disease, chronic obstructive pulmonary disease, and arrhythmia before surgery, and the perioperative management is difficult, with many complications, resulting in high postoperative mortality.

Surprisingly, contrary to the results of a study conducted in the United States [15]. This may be due to differences in specific sociodemographic and clinical characteristics of the samples in the two studies, including predictability of treatment and disease progression. At the same time, body mass index (BMI) was also an independent risk factor for in-hospital mortality in patients undergoing AAAD surgery. Previous studies have shown [16] that overweight and obese patients have a higher risk of postoperative complications, pulmonary complications, and low cardiac output syndrome. A previous literature [17] reported that higher BMI can induce kidney damage in patients after AAD and increase the risk of death, especially in obese patients.

A previous study reported the prognostic value of leukocytes at admission in AAAD patients [18], and the results of this study confirmed this notion. Elevated white blood cell count can reflect the acute inflammatory response of aortic dissection and the degree of aortic injury. The normal structure of the arterial wall is destroyed, the compliance is decreased, and the resistance to blood pressure is weakened, which leads to the occurrence and development of dissection. During cardiopulmonary bypass and deep hypothermic circulatory arrest, the brain, lung, liver, kidney and other organs are injured due to ischemia–reperfusion and inflammatory reaction. This results in increased capillary permeability, tissue edema, and ultimately multiple organ failure, resulting in a significant increase in mortality in AAD patients. Previous studies have shown that about 80.0% of patients with aortic dissection also have hypertension [19]. Hypertension is a major risk factor for dissection rupture and increases the risk of death. The results of this study also verified that hypertension is an independent risk factor for postoperative in-hospital mortality in patients with AAAD. Therefore, it is particularly important to strengthen the standardized treatment of hypertensive patients. At the same time, the perioperative management of high-risk patients was strengthened to improve the prognosis of patients.

Disorders of blood lipid levels and abnormal metabolism are closely related to the risk and prognosis of cardiovascular disease. Low levels of high-density lipoprotein cholesterol and high levels of triglycerides have been proven risk factors for chronic diseases such as coronary heart disease, diabetes, fatty liver, and metabolic syndrome. Blood lipid analysis has a predictive effect on early cardiovascular risk, diabetes, fatty liver, metabolic syndrome, and insulin resistance, which has been confirmed by many studies [20]. Recent studies have shown that abnormal lipid metabolism is closely related to aortic disease [21]. Zhao et al. [22] found that compared with normal control subjects, patients with abdominal aortic aneurysm had lower HDL-C levels and higher LDL-C levels. All the above studies suggest that dyslipidemia plays an important role in aortic disease.

Notably, our study showed that the admission TG/HDL-C ratio was significantly associated with in-hospital mortality. Higher TG/HDL-C levels can lead to a significant increase in in-hospital death, which suggests that patients with aortic dissection who died in hospital had more obvious dyslipidemia than those who survived AAAD. The proportion of HDL-C and TG levels in patients with aortic dissection who died in hospital is seriously imbalanced, which affects endothelial function, enhances oxidative stress, and leads to systemic inflammation. The study of Wilkins et al. [23] suggested that the lower the level of HDL-C, the higher the probability of coronary heart disease, and it is an independent predictor of cardiovascular events in the population. Okanara et al. [24] conducted a follow-up study for nearly 10 years and showed that HDL-C was inversely proportional to its all-cause mortality. The mechanism may be related to low levels of HDL-C are not conducive to the regulation of extracellular matrix reconstruction, cell differentiation and proliferation [25, 26]. A study [27] found that serum TG levels were significantly increased in patients with aortic dissection. Its pathophysiological significance may be related to the increased serum TG level aggravating the impaired endothelial vasodilation function of aortic vessels, thereby leading to the formation of aortic dissection. At the same time, the study found that TG was significantly positively associated with the risk of cardiovascular events and all-cause mortality. With the increase of TG levels, the incidence of ischemic heart disease and ischemic stroke in the research subjects showed a significant upward trend [28]. Therefore, early identification of dyslipidemia chest pain patients and comprehensive strategies such as reasonable blood lipid management, drug prevention, healthy lifestyle adjustment, and exercise intervention can achieve the goals of controlling the occurrence and development of aortic dissection and reducing postoperative complications.

However, compared with the single blood lipid indicators of TG and HDL-C, it cannot fully reflect the overall level of blood lipids. TG/HDL-C ratio, as a combined blood lipid index, can be used as a simple, accessible and reliable hematological index for predicting cardiovascular risk. The TG/HDL ratio has been shown to be a strong predictor of total mortality, coronary heart disease incidence, and cardiovascular mortality. It was not associated with important prognostic variables including age, ethnicity, smoking, hypertension, diabetes, and severity of coronary heart disease [29]. Caselli et al. [30] found that low HDL-C levels and high TG/HDL-C ratios were risk factors for cardiovascular events in patients with coronary heart disease. The ratio of TG/HDL-C can more accurately reflect the comprehensive level of lipid metabolism in patients than the single blood lipid measurement results. Therefore, using the ratio of TG/HDL-C as a predictor of cardiovascular events in coronary heart disease can more accurately predict the residual cardiovascular risk in patients with coronary heart disease. Studies have found that high levels of TG/HDL-C can lead to the development of abdominal aortic aneurysm, and it is positively correlated with the severity of the patient's disease [31]. Consistent with the results of this study, further correlation analysis in this study showed that the TG/HDL-C ratio level was positively associated with in-hospital mortality in AAAD patients. In this study, TG/HDL-C was confirmed to be an independent predictor of mortality risk in AAAD patients. Adding other predictive ratios (such as preoperative monocyte to high-density lipoprotein ratio) using HDL-C as a component predicted postoperative cardiac patients with hospitalization and long-term mortality. Whether it will affect other adverse prognostic outcomes remains to be further confirmed by future studies [32, 33]. Consider that the effect of blood lipids on poor prognosis may be different between different populations. Therefore, we further performed subgroup analysis and found that the ratio of TG/HDL-C was significantly associated with in-hospital mortality in patients with elderly, BMI > 28 kg/m2, and hypertension grade 2 or higher. This reminds us that special attention should be paid to the elderly, BMI > 28 kg/m2 and hypertension grade 2 or above, especially those with higher TG/HDL-C.

There are still some limitations in this study. First, because this study is a single-center observational study. Due to the large differences in patients' clinical characteristics, diagnosis and treatment measures, especially surgical experience, the results of this study need to be verified by a multi-center study. Second, this study only analyzed the TG/HDL-C ratio at admission and within 24 h. Whether serial blood lipid testing after admission has a higher predictive value for the prognosis of aortic dissection remains to be studied. In addition, further long-term follow-up studies are needed to understand the effect of serum TG/HDL-C levels on long-term prognosis.

## Conclusion

In conclusion, this study demonstrates that serum TG/HDL-C levels are a potential clinical prognostic factor in patients after AAAD. Serum TG/HDL-C levels were positively associated with in-hospital mortality in patients after AAAD in the Chinese population. Preoperative risk assessment should pay special attention not only to those high-risk groups, but also to patients with dyslipidemia. Early clinical intervention may be effective in reducing the risk of in-hospital mortality.

## Data Availability

The datasets generated and/or analysed during the current study are not publicly available due to individual privacy, but are available from the corresponding author on reasonable request.

## Abbreviations

AAD: Acute aortic dissection
CTA: Computed tomography angiography
IQR: Interquartile range

## References

[1] Tang X, Lu K, Liu X, Jin D, Jiang W, Wang J, Zhong Y, Wei C, Wang Y, Gao P (2021). Incidence and survival of aortic dissection in urban China: results from the national insurance claims for epidemiological research (NICER) Study. Lancet Regional Health Western Pacific.

[2] Gudbjartsson T, Ahlsson A, Geirsson A, Gunn J, Hjortdal V, Jeppsson A, Mennander A, Zindovic I, Olsson C (2020). Acute type A aortic dissection - a review. Scand Cardiovasc J: SCJ.

[3] Uehara K, Matsuda H, Matsuo J, Inoue Y, Omura A, Seike Y, Sasaki H, Kobayashi J (2018). Acute type A aortic dissection repair in younger patients. J Card Surg.

[4] Reutersberg B, Salvermoser M, Trenner M, Geisbüsch S, Zimmermann A, Eckstein HH, Kuehnl A (2019). Hospital incidence and in-hospital mortality of surgically and interventionally treated aortic dissections: secondary data analysis of the nationwide german diagnosis-related group statistics from 2006 to 2014. J Am Heart Assoc.

[5] Ren Y, Huang S, Li Q, Liu C, Li L, Tan J, Zou K, Sun X (2021). Prognostic factors and prediction models for acute aortic dissection: a systematic review. BMJ Open.

[6] Li M, Xu S, Yan Y, Wang H, Zheng J, Li Y, Zhang Y, Hao J, Deng C, Zheng X (2021). Association of biomarkers related to preoperative inflammatory and coagulation with postoperative in-hospital deaths in patients with type A acute aortic dissection. Sci Rep.

[7] Chen Z, Chen G, Qin H, Cai Z, Huang J, Chen H, Wu W, Chen Z, Wu S, Chen Y (2020). Higher triglyceride to high-density lipoprotein cholesterol ratio increases cardiovascular risk: 10-year prospective study in a cohort of Chinese adults. J Diabetes Investig.

[8] Liu X, Su X, Zeng H (2016). Impact of admission serum total cholesterol level on in-hospital mortality in patients with acute aortic dissection. Pak J Med Sci.

[9] Zhou Y, Yang G, He H, Pan X, Peng W, Chai X (2020). Triglyceride/high-density lipoprotein cholesterol ratio is associated with in-hospital mortality in acute type B aortic dissection. Biomed Res Int.

[10] Erbel R, Aboyans V, Boileau C, Bossone E, Bartolomeo RD, Eggebrecht H, Evangelista A, Falk V, Frank H, Gaemperli O et al. 2014 ESC Guidelines on the diagnosis and treatment of aortic diseases: Document covering acute and chronic aortic diseases of the thoracic and abdominal aorta of the adult. The Task Force for the Diagnosis and Treatment of Aortic Diseases of the European Society of Cardiology (ESC). Eur Heart J. 2014;35(41):2873–2926. 10.1093/eurheartj/ehu281 25173340

[11] Pagni S, Ganzel BL, Trivedi JR, Singh R, Mascio CE, Austin EH, Slaughter MS, Williams ML (2013). Early and midterm outcomes following surgery for acute type A aortic dissection. J Card Surg.

[12] Bashir M, Harky A, Shaw M, Adams B, Oo A (2019). Type A aortic dissection in patients over the age of seventy in the UK. J Card Surg.

[13] Lin HS, Watts JN, Peel NM, Hubbard RE (2016). Frailty and post-operative outcomes in older surgical patients: a systematic review. BMC Geriatr.

[14] Evangelista A, Isselbacher EM, Bossone E, Gleason TG, Eusanio MD, Sechtem U, Ehrlich MP, Trimarchi S, Braverman AC, Myrmel T (2018). Insights from the international registry of acute aortic dissection: a 20-year experience of collaborative clinical research. Circulation.

[15] Kreibich M, Rylski B, Bavaria JE, Branchetti E, Dohle D, Moeller P, Vallabhajosyula P, Szeto WY, Desai ND (2018). Outcome after operation for aortic dissection type A in morbidly obese patients. Ann Thorac Surg.

[16] Lio A, Bovio E, Nicolò F, Saitto G, Scafuri A, Bassano C, Chiariello L, Ruvolo G (2019). Influence of body mass index on outcomes of patients undergoing surgery for acute aortic dissection: a propensity-matched analysis. Tex Heart Inst J.

[17] Liu T, Fu Y, Liu J, Liu Y, Zhu J, Sun L, Gong M, Dong R, Zhang H (2021). Body mass index is an independent predictor of acute kidney injury after urgent aortic arch surgery for acute DeBakey Type I aortic dissection. J Cardiothorac Surg.

[18] Fan X, Huang B, Lu H, Zhao Z, Lu Z, Yang Y, Zhang S, Hui R (2015). Impact of admission white blood cell count on short- and long-term mortality in patients with type A acute aortic dissection: an observational study. Medicine (Baltimore).

[19] Zhao L, Chai Y, Li Z (2017). Clinical features and prognosis of patients with acute aortic dissection in China. J Int Med Res.

[20] Kiyosue A. Nonfasting TG/HDL-C ratio seems a good predictor of MACE in CAD patients with statin therapy: could it be a treatment target? J Cardiol. 2018;71(1):8–9. 10.1016/j.jjcc.2017.09.001 29033056

[21] He BM, Chai XP, He ZB, Guo T, Peng W, Peng ZY (2014). HDL quantity and function are potential therapeutic targets for abdominal aortic aneurysm. Int J Cardiol.

[22] Zhao L, Jin H, Yang B, Zhang S, Han S, Yin F, Feng Y (2016). Correlation between ABCA1 gene polymorphism and aopA-I and HDL-C in abdominal aortic aneurysm. Med Sci Monit: Int Med J Exp Clin Res.

[23] Wilkins JT, Ning H, Stone NJ, Criqui MH, Zhao L, Greenland P, Lloyd-Jones DM (2014). Coronary heart disease risks associated with high levels of HDL cholesterol. J Am Heart Assoc.

[24] Okamura T, Hayakawa T, Kadowaki T, Kita Y, Okayama A, Ueshima H (2006). The inverse relationship between serum high-density lipoprotein cholesterol level and all-cause mortality in a 9.6-year follow-up study in the Japanese general population. Atherosclerosis.

[25] Prasad M, Sara J, Widmer RJ, Lennon R, Lerman LO, Lerman A (2019). Triglyceride and triglyceride/ HDL (high density lipoprotein) ratio predict major adverse cardiovascular outcomes in women with non-obstructive coronary artery disease. J Am Heart Assoc.

[26] Ottosson F, Emami Khoonsari P, Gerl MJ, Simons K, Melander O, Fernandez C (2021). A plasma lipid signature predicts incident coronary artery disease. Int J Cardiol.

[27] Uruska A, Zozulinska-Ziolkiewicz D, Niedzwiecki P, Pietrzak M, Wierusz-Wysocka B (2018). TG/HDL-C ratio and visceral adiposity index may be useful in assessment of insulin resistance in adults with type 1 diabetes in clinical practice. J Clin Lipidol.

[28] Bittner V, Johnson BD, Zineh I, Rogers WJ, Vido D, Marroquin OC, Bairey-Merz CN, Sopko G (2009). The triglyceride/high-density lipoprotein cholesterol ratio predicts all-cause mortality in women with suspected myocardial ischemia: a report from the Women's Ischemia Syndrome Evaluation (WISE). Am Heart J.

[29] Drexel H, Aczel S, Marte T, Benzer W, Langer P, Moll W, Saely CH (2005). Is atherosclerosis in diabetes and impaired fasting glucose driven by elevated LDL cholesterol or by decreased HDL cholesterol?. Diabetes Care.

[30] Caselli C, De Caterina R, Smit JM, Campolo J, El Mahdiui M, Ragusa R, Clemente A, Sampietro T, Clerico A, Liga R (2021). Triglycerides and low HDL cholesterol predict coronary heart disease risk in patients with stable angina. Sci Rep.

[31] Ma W, Cui C, Feng S, Li G, Han G, Hu Y, Li X, Lv J, Liu C, Jin F (2019). Serum uric acid and triglycerides in chinese patients with newly diagnosed Moyamoya disease: a cross-sectional study. Biomed Res Int.

[32] Tekkesin AI, Hayiroglu MI, Zehir R, et al. The use of monocyte to HDL ratio to predictpostoperative atrial fibrillation after aortocoronary bypass graft surgery. Northern Clin Istanbul, 2017;4(2). 10.14744/nci.2017.53315 PMC5613262 28971172

[33] Avci II, Sahin I, Gungor B (2018). Association of monocyte to high-density lipoprotein ratio with bare-metal stent restenosis in STEMI patients treated with primary PCI. North Clin Istanb.

